# The Influence of Self-Referential Processing on Attentional Orienting in Frontoparietal Networks

**DOI:** 10.3389/fnhum.2018.00199

**Published:** 2018-05-15

**Authors:** Shuo Zhao, Shota Uono, Chunlin Li, Sayaka Yoshimura, Motomi Toichi

**Affiliations:** ^1^Faculty of Human Health Science, Graduate School of Medicine, Kyoto University, Kyoto, Japan; ^2^Organization for Promoting Neurodevelopmental Disorder Research, Kyoto, Japan; ^3^Department of Neurodevelopmental Psychiatry, Habilitation and Rehabilitation, Graduate School of Medicine, Kyoto University, Kyoto, Japan; ^4^School of Biomedical Engineering, Capital Medical University, Beijing, China

**Keywords:** self-referential processing, attentional orienting, dorsal frontoparietal network, ventral frontoparietal network, cortical midline structures

## Abstract

Self-referential processing refers to the processing of information relevant to oneself and plays an important role in cognition. Behavioral studies have shown that directional cue stimuli have a qualitatively different function during attentional orienting after presentation of the cue associated with the self. However, it is necessary to determine how neural activity is influenced by self-referential processing during attentional orienting. The present study involved establishing an association between non-predictive arrow cues and the “self” during a training task and then investigating the influence of self-referential processing on neural activity during attentional orienting. Enhanced neural activity was observed in cortical midline structures (CMS) during the use of self- vs. neutral-arrow cues, which suggests that the arrow associated with the “self” triggered self-referential processing during attentional orienting due to the experiences of the participant in the training task. Comparison of obtained under the incongruent and congruent conditions revealed a qualitative difference in neural activities between the self- and neutral-arrow cues associated with attentional orienting. Furthermore, when the neutral-arrow cue was treated as a baseline condition, neural activity was reduced in the frontoparietal attention networks by self-referential processing under the incongruent condition, but it was enhanced under the congruent condition. Thus, the stimulus modulated subsequent attentional neural processes after being associated with the self as a cue, which indicates that this process may be triggered by self-reference to automatically and effectively capture information. Our findings extend those of previous behavioral studies of neural activity, suggesting that directional cues were qualitatively influenced by self-referential processing, and showed different functions during attentional orienting. Moreover, the present study provides important evidence of how self-referential processing affects attentional orienting in the frontoparietal network.

**Highlights**

- Enhanced activity was observed in CMS due to self-referential processing.
- The influence of self-referential processing differed in the frontoparietal network.
- Activity was enhanced by self-referential processing under the congruent condition.
- Activity was reduced by self-referential processing under the incongruent condition.

## Introduction

Humans commonly exhibit biased responses such that they preferentially encode information relevant to themselves compared to information relevant to others (e.g., Rogers et al., 1977; Keyes and Brady, 2010; Keyes, 2012; Nakao et al., 2012). For example, when people are asked to remember specific information, the recall rates are better when such information is related to themselves than to other people (Rogers et al., 1977). More recent studies have reported the faster classification of self-faces compared to the faces of other people when participants were asked to classify faces as self, friend, or stranger (Keyes and Brady, 2010; Keyes, 2012). This phenomenon is known as the self-referential effect and has been observed in children and young and older adults (e.g., Sui and Zhu, 2005; Gutchess et al., 2007, 2010, 2015; Cunningham et al., 2014). The advantages associated with self-referential processing may also be relevant when information is assigned a new association with the self. Previous studies (Sui et al., 2015; Sui and Humphreys, 2017) have indicated that self-reference operates as an integrative mechanism during information processing to either enhance or disrupt the performance of tasks related to perception (Sui et al., 2012), memory (Kelley et al., 2002), and decision-making (Sui and Humphreys, 2013) when self-referential information is associated with stimuli. Thus, it is important to understand the mechanisms underlying the manner in which self-reference influences subsequent stages of information processing.

Self-referential processing may also aid in the preferential analysis of information during attentional orienting (Sui et al., 2009; Zhao et al., 2015). To avoid the familiarity effect and investigate the influence of self-referential processing on attentional orienting, Zhao et al. (2015) suggested that a directional stimulus (e.g., a red arrow) is regarded as a self-referential cue, whereas a different directional stimulus (e.g., a green arrow) is associated with a strange person and is regarded as the other-referential cue in a training task. Then, in a cueing task, participants were required to indicate whether sound targets (voice or tone) were presented at the left or the right location (i.e., a localization task) was conducted. Zhao et al. (2015) showed that self- but not other-referential arrow cues induced a pattern of attentional orienting that was similar to that elicited by gaze (Zhao et al., 2014). This finding suggests that directional cues, such as arrows, have the ability to manifest a qualitatively different function during attentional orienting due to self-referential processing. However, this finding is supported by only behavioral evidence; thus, it is necessary to determine the manner in which neural activity is influenced by self-referential processing during attentional orienting to arrow cues.

Although no previous study has directly investigated the neural correlates of the effects of self-referential processing under the attentional cueing paradigm, previous studies have demonstrated separate neural correlates for self-referential processing and attention orienting. Activity in cortical midline structures (CMSs), such as the anterior cingulate cortex (ACC)/ventromedial prefrontal cortex (vmPFC), has been shown to be associated with self-referential processing (for a review, see Northoff and Bermpohl, 2004). For example, Sui et al. (2013) used geometric shapes to establish an association between “self” and “others,” and immediately implemented a matching task involving judgments of whether paired shapes and words were matched or mismatched. The results revealed increased activity to self-associated pairs in the vmPFC. Several aspects of attention orienting are involved in the cortical activity of two attentional networks (e.g., Zhao et al., 2017). The dorsal frontoparietal network has been associated with orienting or attending to a cued target in cueing paradigms, as well as being involved in reorienting attention to an uncued target, while the ventral frontoparietal network is thought to be responsible only for reorienting attention (Corbetta et al., 2008; Corbetta and Shulman, 2011).

Combining behavioral and neural evidence, we suggest that the neural activity of attentional orienting to arrow cues may be qualitatively influenced by self-referential processing. Given that Zhao et al. (2015) reported that self-referential, but not other-referential, arrow cues could induce a pattern of attentional orienting that was similar to that elicited by gaze (Zhao et al., 2014), it is possible that the neural pattern of arrow cues influenced by self-referential processing is similar to the effect of gaze on attentional orienting. Engell et al. (2010) showed a qualitative difference between the neural pattern of attentional orienting in response to gaze vs. arrow cues. Compared with arrow cues, which induced increased activity, no difference in the responses to incongruent vs. congruent gaze cues were induced in the dorsal and ventral frontoparietal networks. Thus, if different patterns of neural activity were not observed between incongruent vs. congruent conditions when using a self-arrow as a cue, then self-referential processing would have induced qualitatively different types of attentional processing.

To investigate how the neural activity underlying attentional orienting induced by arrow cues was influenced by self-referential processing, the present study manipulated the self-referentiality of arrow stimuli in a training task, in which participants were trained to associate two arrows (e.g., red and green arrows) with the words “self” and “other.” Subsequently, in the attentional cueing task, we used a self-referential and neutral arrow cue to direct attention toward the right or left of a screen, and a target was presented at either the cued or opposite location. Participants were instructed to indicate whether the target letter was presented at the left or the right location. This behavioral task has been successfully performed with various types of directional cue (e.g., arrow and gaze see Engell et al., 2010); thus, comparisons between self- and neutral-arrow cues are less likely to be confounded by behavioral differences. Because enhanced neural activity of CMSs was observed with the use of self- vs. neutral-arrow cues in the attentional cueing task, we proposed that the arrow associated with “self” may trigger self-referential processing during attentional orienting due to the experiences of the participant in the training task. Thus, we assessed neural activities within the dorsal and ventral frontoparietal networks to examine whether qualitatively different types of attentional processing between self- and neutral-arrow cues emerged through self-referential processing.

## Materials and Methods

### Participants

The present study was approved by the local ethics committee of Capital Medical University, Beijing, China. No foreseeable risks to the participants were present, and no information that could personally identify the individuals was collected. All participants provided written informed consent and background information, and all procedures complied with the ethical standards of the 1964 Declaration of Helsinki regarding the treatment of human participants in research. In total, 24 volunteers (17 women and seven men; mean ± SD age: 21.42 ± 1.18 years) participated in this study. All participants were right-handed as assessed by the Edinburgh Handedness Inventory (Oldfield, 1971) and had normal or corrected-to-normal visual and auditory acuity.

### Stimuli

**Figure 1:**
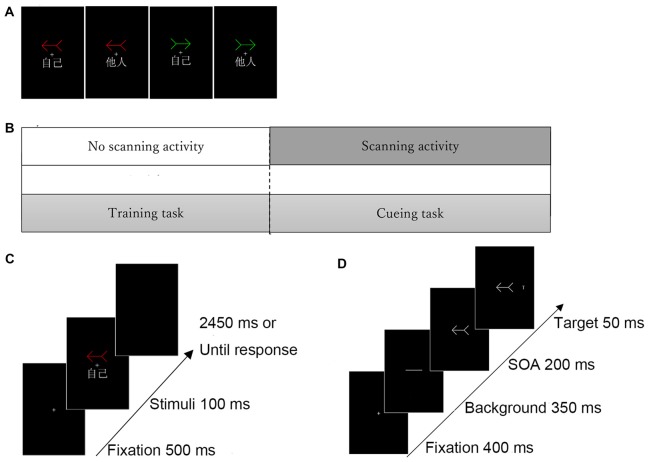
Experimental task structure. **(A)** Examples of self- and other-arrow pair stimuli. **(B)** Procedure of the experimental task. **(C)** Illustration of stimulus presentation in the **(C)** training task and **(D)** cueing task. Three different colored arrows (i.e., red, green and white) were included. Only two arrows (red and green) were associated with the “self” or “other” words in the training task and the white arrow was used as a neutral-arrow in the cueing task but not in the training task.

Figure 1A illustrates the stimuli used in the training task. A red, green, or white arrow (5.2° wide × 3.2° high) was presented above the fixation cross, whereas the word “self” (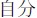) or “other” (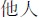) (2.9° wide × 2.0° high) was displayed below the fixation cross. In the cueing task, the stimuli (i.e., the red, green, and white arrows) were the same as those in the training task. The letter “T” (0.6° wide and 0.6° high) was presented 5.2° to the left or right side of the center of the screen as a target stimulus.

### Apparatus

All stimuli were generated on a Windows computer and presented to the participants via a custom-built magnet-compatible audio–visual system during magnetic resonance imaging (MRI) scans. Presentation software (ver. 10.2; Neurobehavioral Systems) was used to generate the visual stimuli on a Windows computer, and participants viewed the visual stimuli on a back-projection screen and generated their responses using a keypad (Current Designs Inc.; Philadelphia, PA, USA).

### Procedure

Two tasks were involved in the present experiment. First, the participants were trained to associate two arrows (e.g., one red and one green) with the words “self” and “other” in the training task, and the self-referential arrow was then used as a self-cue and a novel arrow (i.e., white) was used as a neutral cue; however, the other-referential arrow was not implemented in the cueing task. The cueing blocks were initiated following the completion of the training block; all participants performed one block of the training task and four blocks of the cueing task (Figure 1B).

### Training Task

The participants were trained to develop an association between self- or other-referential information and two arrows (Figure 1A). Each arrow was associated with the word “self” or “other.” For example, a red and a green arrow were associated with “self” and “other,” respectively. Another arrow (e.g., a white arrow) was used as a neutral-arrow in the subsequent cueing task. Thus, one of six different patterns of associations were implemented for each participant in the training task (i.e., a red arrow for “self” and a green arrow for “other,” a red arrow for “self” and a white arrow for “other,” a green arrow for “self” and a red arrow for “other,” a green arrow for “self” and a white arrow for “other,” a white arrow for “self” and a red arrow for “other,” and a white arrow for “self” and a green arrow for “other”). These patterns were counterbalanced across participants.

Each trial began with a display consisting of a central fixation cross that was presented for 500 ms in the center of the screen. Then, the training stimuli were presented for 100 ms in conjunction with a colored arrow (e.g., red or green) and the assigned or unassigned word (“self” or “other”), irrespective of the direction of the arrows. The participants were instructed to only respond when the relationship between the colored arrow and the assigned word was correct by pressing a button as quickly and accurately as possible. Although a total of four pairs were included (e.g., red arrow-self, red arrow-other, green arrow-self, green arrow-other), participants were requested to respond only to two correct pairs (e.g., red arrow-self and green arrow-other). The cue remained on the screen until the participant responded or 3050 ms had elapsed (Figure 1C). Each participant performed one block of 280 trials, in which each stimulus was presented during 70 trials and all combinations of arrows and words occurred in equal proportions in a randomized order.

### Cueing Task

An example of the procedure in the cueing task is shown in Figure 1D. Each trial began with a display consisting of a fixation cross that was presented for 400 ms in the center of the screen. Then, a transverse white line as neutral stimulus was presented for 350 ms, and the cue stimulus pointing right or left (e.g., a colored arrow associated with “self”) was subsequently presented. The stimulus onset asynchrony (SOA) between the target and cue was fixed at 200 ms. Finally, the target letter “T” appeared to the left or the right side of the cue stimulus for 50 ms. The participants were asked to respond as quickly as possible when a target appeared on the left or the right side by pressing the corresponding key on the switch keypad using their dominant index or middle finger, respectively; reaction times (RTs) were measured in each trial. The cue remained on the screen until the participant responded or 1000 ms had elapsed. The targets appeared randomly at the cued location in 50% of the trials. The participants were told that the cues were uninformative with respect to the potential locations of the subsequent target and were instructed to fixate on the center of the screen in each trial.

The functional MRI (fMRI) analysis was based on a within-subjects 2 × 2 factorial design with the cue condition (self- or neutral-arrow) and congruence condition (congruent or incongruent) as repeated factors; an arrow associated with “self” was used as a self-relevant arrow cue (e.g., red arrow) and the other arrow, which was not presented in the training task and was not associated with a specific word, was used as a neutral-arrow cue (e.g., a white arrow). In total, 60 trials were performed under each condition. Four blocks were included in the cueing task. Each block had 120 trials, with 60 cueing and 60 rest trials. The present experimental design was based on a mixed block/event-related paradigm that allows for the more complete utilization of the blood-oxygen-level-dependent (BOLD) signal which, in turn, enables a deeper interpretation of how brain regions function on multiple timescales (Petersen and Dubis, 2012). As in previous studies (Friston et al., 1999; Yan et al., 2017), we presented alternating blocks of experimental trials with the cue condition as well as blocks for baseline measures. The congruence trials were presented in a pseudorandom event-related distribution within the experimental blocks.

### MRI Acquisition

MR images were acquired using a 3.0-T Trio Tim Scanner-vision (Siemens; Erlangen, Germany). A whole-body MRI system was employed to measure activation with a head coil. The functional images consisted of 33 consecutive slices parallel to the plane of the anterior–posterior commissure and covered the whole brain. A T2*-weighted gradient-echo planar imaging (EPI) sequence with the following parameters was used: TR = 2000 ms, TE = 30 ms, flip angle = 90°, field of view = 220 × 220 mm, matrix size = 64 × 64, and voxel size = 3.4 × 3.4 × 3.5 mm^3^. Excluding the most-inferior parts of the cerebellum, most of the brain regions, including the entire temporal cortex, were imaged. Furthermore, high-resolution isotropic T1-weighted images were acquired using the following parameters: TR = 1900 ms, TE = 2.52 ms, flip angle = 9°, field of view = 250 × 250 mm, 176 sagittal slices, and voxel size = 1 × 1 × 1 mm^3^.

### Data Analysis

#### Behavioral Data Analysis

All data were analyzed using SPSS software (ver. 21.0; IBM Corp.). To assess the strength of the association between arrow color and self- or other-referential words using a cut-off of 10% errors in any block, we measured total error rates (TERs), including omission and commission errors in the training task. Consistent with a previous study (Zhao et al., 2015), at least 90% of the trials were required to be correct in the block, and RTs shorter than 100 ms or longer than 1000 ms were excluded from the RT analysis (1.19% of the trials). The difference in mean accuracy and RTs between self- and other-arrow stimuli was calculated for each participant. For this analysis, we used paired *t-*tests.

In the cueing task, the mean RT of the correct responses was calculated for each condition and each participant, excluding incorrect responses (1.20% of the trials) and RTs shorter than 100 ms or longer than 1000 ms were excluded from the RT analysis (6.15% of the trials). Additionally, the present study assessed whether a speed/accuracy trade-off occurred. Given that the rates of incorrect responses were so low and there was a floor effect for the accuracy scores, we did not further analyze the error data. Next, the mean RTs were analyzed using a two-way analysis of variance (ANOVA) with cue (self- and neutral-arrow) and congruence (congruent and incongruent) as the within-subject factors. Significant interactions were analyzed further with follow-up simple main effect analysis (Kirk, 1995).

#### Image Data Analysis

The imaging data from the cueing task were analyzed in the present study. Data pre-processing and statistical analyses were performed using the Statistical Parametric Mapping computer package (SPM12; Wellcome Department of Cognitive Neurology, London, UK^1^) implemented in MATLAB 2013b (MathWorks). First, to correct for head movement, functional images of each run were realigned using the first scan as a reference. The movement parameters generated during spatial realignment showed that the subjects moved less than 2 mm during the course of the trial. Then, the T1 anatomical image was coregistered to the first scan of the functional images. Subsequently, all anatomical and functional images were normalized to Montreal Neurological Institute (MNI) space using the anatomical image-based unified segmentation-spatial normalization approach (Ashburner and Friston, 2005); Finally, these spatially normalized functional images were resampled to a voxel size of 2 × 2 × 2 and smoothed with an 8-mm full-width-at-half-maximum (FWHM) Gaussian kernel at half-maximum to compensate for anatomical variability among participants.

We conducted random-effects analyses to identify voxels with significant activation at the population level (Holmes and Friston, 1998). First, a single-subject analysis (Friston et al., 1995) was performed. For each condition, The BOLD response was modeled as the neural activity and was convolved with a canonical hemeodynamic response function (HRF) that yielded regressors in a general linear model (GLM). We used a high-pass filter with a cut-off period of 128-s to eliminate the artifactual low-frequency trend. Global scaling was conducted to correct for global fluctuation related to motion artifacts. Serial autocorrelation was assumed to follow a first-order autoregressive (AR[1]) model, which was estimated from the pooled active voxels with a restricted maximum likelihood procedure and used to whiten the data and design the matrix (Friston et al., 2002).

The contrast images from the first-level analyses of all subjects were then used for the second-level group statistics. First, the data for each participant were best-fitted (least square fit) at every voxel using a linear combination of the effects of interest. These included delta functions representing the onsets of the four conditions given by the convolving of the 2 × 2 factorial design (cue [self- and neutral-arrow] × congruence [congruent and incongruent]) with the SPM12 HRF. Second, based on the behavioral results, a 2 × 2 (cue × congruence) factorial ANOVA was performed to investigate the relationship between the behavioral results and brain activation. Based on methods analysis (Woo et al., 2014; Eklund et al., 2016), voxels were identified as significantly activated if they reached a cluster-level threshold of *p* < 0.05 (family-wise error [FWE]-corrected for multiple comparisons) and a voxel-level threshold of *p* < 0.001 (uncorrected for multiple comparisons at the whole-brain level), which were used to protect against false-positive activations. The peak voxels of clusters exhibiting reliable effects are reported in the stereotactic coordinates of MNI. a priori hypotheses regarding the neural activity associated with self-referential processing in the ACC/vmPFC and precuneus/PCC and the influence of self-referential processing on attentional orienting in the dorsal and ventral frontoparietal networks were stated.

Based on anatomical masks outlined using the WFU PickAtlas tool, a small volume correction (SVC) procedure was employed separately to the* a priori* regions of interest (ROIs): the bilateral anatomical structures in the CMSs, such as the ACC/vmPFC (Brodmann area [BA] 10/24/32), the precuneus/PCC (BA 7/23/31), and the dorsal and ventral frontoparietal network (e.g., Thiel et al., 2004; Doricchi et al., 2010; Yan et al., 2015; Zhao et al., 2017), such as the FEF (BA8), the VFG (BA 44/45/47), and the TPJ (BA 22/39/40). Consistent with the whole-brain level analysis, SVC analysis was performed with a cluster-level threshold of *p* < 0.05 (FWE-corrected for multiple comparisons) and a voxel-level threshold of *p* < 0.001 (uncorrected for multiple comparisons). To examine whether the significant brain activities were specific to those in the regions involved in self-referential processing or attentional processing, we used a control analysis with a relatively liberal threshold (voxel-level threshold of *p* < 0.001 uncorrected with a minimum cluster-level threshold of five voxels) at the whole-brain level. Furthermore, to assess the relationship between the behavioral response and significant brain activation during self-referential processing, we calculated Pearson’s correlation coefficients between the RT under the self-arrow condition and the beta values in an 8-mm radius sphere centered on the peak voxel of activation.

Finally, to quantify neural responses associated with the influence of self-referential processing on attentional orienting, beta values for the self- and neutral-arrows under the congruent and incongruent conditions were extracted and averaged across voxels in the given ROIs using spheres with a radius of 8 mm. The means of the beta values between conditions were compared with a 2 (cue: self- and neutral-arrow) × 2 (congruence: congruent and incongruent) repeated-measures ANOVA. Because only two variables were included in each independent condition, a follow-up simple main effect analysis was conducted (*p* < 0.05) if a two-way interaction was significant. All statistics were calculated using SPSS software (ver. 21.0).

## Results

### Behavioral Results

#### Training Task

The TERs for all 24 participants were less than 10% in the training task (mean ± SD: 2.3 ± 1.18%); all data were analyzed. The participants responded significantly more quickly to the arrow associated with “self” than to the arrow associated with “other” (551.3 vs. 603.1 ms; *t*_(23)_ = 9.30, *p* < 0.001), which indicates that self-referential information has a higher processing priority than other-referential information. Accuracy was also significantly higher in response to the arrow associated with “self” than to the arrow associated with “other” (99.8 vs. 99.6%; *t*_(23)_ = 2.326, *p* = 0.029). These results indicate that the associations between the words (“self” and “other”) and arrow cues were established.

#### Cueing Task

**Figure 2:**
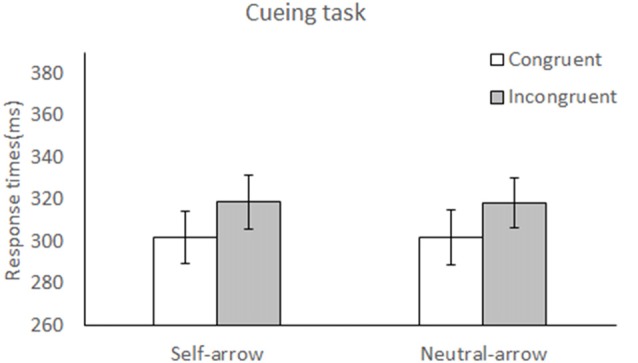
Reaction times (RTs) during attentional orienting in the cueing task. Mean RT (± SE) under the congruent and incongruent conditions as a function of cue condition (self- or neutral-arrow). The results revealed a main effect of congruence, but not cue, which indicates a delayed response under the incongruent compared to the congruent condition. However, no significant interaction was observed between cue and congruence. Statistically significant at alpha = 0.05.

The mean RTs under each condition are listed in Table 1, and the mean differences in RTs between the congruent and incongruent conditions for the self- and neutral-arrow cues are shown in Figure 2. A two-factor repeated-measures ANOVA with cue (self- and neutral-arrow) and congruence (congruent and incongruent) as the within-subject factors was used to analyze RTs and revealed a main effect of congruence (*F*_(1,23)_ = 13.55, *p* = 0.001, $\eta_{\text{p}}^{2}$ = 0.37) but not cue (*F*_(1,23)_ = 0.005, *p* = 0.94, $\eta_{\text{p}}^{2}$ < 0.001), which indicates that there was a delayed response under the incongruent compared to the congruent condition. However, no significant interaction was observed between cue and congruence (*F*_(1,23)_ = 0.004, *p* = 0.95, $\eta_{\text{p}}^{2}$ < 0.001).

**Table 1:** Mean reaction time (RT), SD and percent errors (%E) as a function of cue and congruence.

Cue	Congruence					
	Congruent			Incongruent		
	*M*	*SD*	%E	*M*	*SD*	%E
Self-arrow	302.2	60.5	1.9	318.7	62.9	0.9
Neutral-arrow	302.2	64.0	0.5	318.4	58.2	2.0

#### fMRI Results

Next, the patterns of brain activation associated with self-referential processing and attentional orienting were investigated. The primary analysis was performed with a 2 (cue conditions [self- and neutral-arrow]) × 2 (congruence conditions [congruent and incongruent]) repeated-measures ANOVA.

#### Main Effects of Cue and Congruence

**Figure 3:**
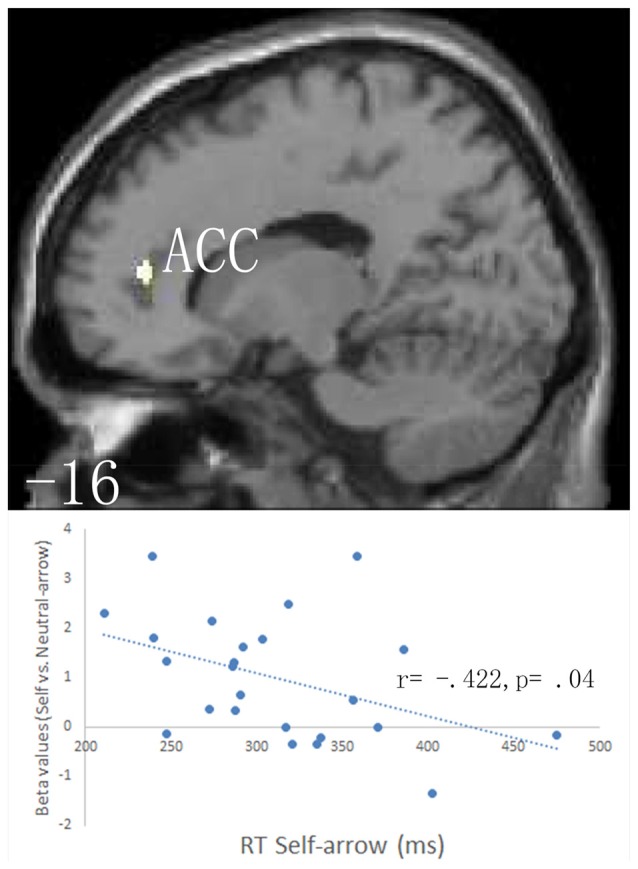
Based on anatomical masks outlined using the WFU PickAtlas tool, a small volume correction (SVC) procedure was employed separately to the* a priori* regions of interest: the bilateral anatomical structures in the anterior cingulate cortex (ACC)/ventromedial prefrontal cortex (vmPFC; BA 10/24/32), the precuneus/posterior cingulate cortex (PCC; BA 7/23/31), the frontal eye field (FEF; BA8), the ventral frontal gyrus (VFG; BA 44/45/47), and the left temporoparietal junction (TPJ; BA 22/39/40). The upper parts of the figure shows SVC analysis of responses to the self- vs. neutral-arrow conditions, which revealed significant activation only in the left ACC based on an anatomical mask; a cluster-level threshold of *p* < 0.05 (FWE-corrected) with a voxel-level threshold of *p* < 0.001 (uncorrected). The lower part of the figure shows the correlation between the behavioral RTs under the self-arrow condition and beta values for the self- vs. neutral-arrow in an 8-mm radius sphere centered on the peak voxel of activation in the left ACC.

An anatomical region-based SVC analysis revealed significant activation in the left limbic lobe, including the ACC when the self- vs. neutral-arrow was presented (Figure 3, Table 2); however, no significant activity was shown in the whole-brain analysis. These results are consistent with those of previous studies (for reviews, see Northoff and Bermpohl, 2004; Schmitz and Johnson, 2007) and indicate that the activity in the ACC was associated with the processing of self-referential information. When using a control analysis at the whole-brain level, significant activation was only found in the CMS, including the bilateral anterior cingulate and right precuneus (Supplementary Table S1). This finding indicated that the effect of the self- vs. neutral-arrow was restricted to regions involved in self-referential processing. Moreover, a significant correlation was found between the RT under the self-arrow condition and differential activity for self- vs. neutral-arrows in the left ACC (*r* = −0.422, *p* = 0.04), indicating that the RT in response to the self-arrow was facilitated by increased activation of contrasting self- and neutral-arrows in the left ACC (Figure 3). Thus, the association between “self” words and the arrow cues was established. An evaluation of differences between the congruence conditions did not reveal any significant activity in either the whole-brain or SVC analysis.

**Table 2:** Main effects of cue condition: self-arrow > neutral-arrow.

Side	Area	Region	BA	Coordinates			Z-value	*P* (FWE) (cluster level)	*P* (FWE) (peak level)	*P* (uncorr) (peak level)	Cluster size
				*x*	*y*	*z*					
Small volume corrections analysis
L	Limbic	Anterior cingulate	32	−16	44	12	3.78	0.043	0.006	0.000	5

*BA, Brodmann area; L, Left; R, Right; FWE, family-wise error; a cluster level at the threshold of *p* < 0.05 (FWE corrected) with the voxel-level at the threshold of *p* < 0.001 (uncorrected). Cluster size is in voxels; voxel size is 2 × 2 × 2 mm^3^*.

#### Interaction of the Cue and Congruence Conditions

**Figure 4:**
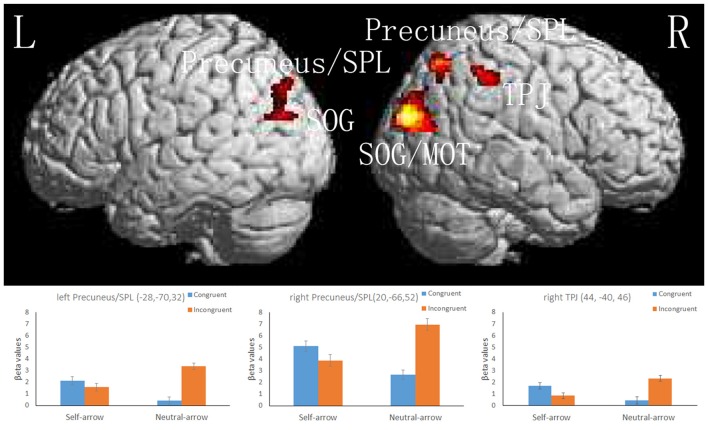
The upper part of the figure shows significant activity in the bilateral superior occipital gyrus (SOG) and superior parietal lobule (SPL), left middle occipital gyrus (MOG), and right TPJ, as revealed by a whole-brain analysis of responses to the interaction between the cue and congruence conditions; a cluster-level threshold of *p* < 0.05 (FWE-corrected) with a voxel-level of threshold of *p* < 0.001 (uncorrected). The lower part of the figure shows the mean beta values (± SE) in the bilateral SPL and right TPJ; these areas are overlaid on the mean normalized structural magnetic resonance imaging (MRI) scans from all participants in the present study.

A 2 (cue: self- and neutral-arrow) × 2 (congruence: congruent and incongruent) ANOVA was performed to investigate the influence of self-referential processing on the attentional orienting networks. The whole-brain analysis revealed significant interaction effects in several visual association areas: the bilateral occipital lobe, including the superior occipital gyrus (SOG); the left temporal lobe, including the middle occipital gyrus (MOG); and the frontoparietal networks, including in the bilateral parietal lobe in regions such as the precuneus/SPL and in the right parietal lobe in the TPJ (Figure 4, Table 3). There was no significant activity in either the FEF or VFG based on the SVC analysis. Moreover, when using a control analysis (an intensity threshold of *p* < 0.001 uncorrected with a minimum spatial extent threshold of five voxels) in the whole-brain level analysis, the results were consistent with the previous analysis (spatial extent threshold of *p* < 0.05 FWE with an intensity threshold of *p* < 0.001 uncorrected). The results indicated differential activities among the bilateral occipital lobe, right paracentral lobule, bilateral precuneus/SPL, right TPJ and IFG, and right SFG/MFG (Supplementary Table S2). Except for the right paracentral lobule, associated with motor function, the other areas have been shown to be closely associated with attentional processing (e.g., Hietanen et al., 2006; Engell et al., 2010; Li et al., 2012).

**Table 3:** Interactions between cue and congruence conditions.

Side	Area	Region	BA	Coordinates			Z-value	*P* (FWE) (cluster level)	*P* (FWE) (peak level)	*P* (uncorr) (peak level)	Cluster size
				*x*	*y*	*z*					
Exploratory whole-brain analysis
R	Occipital	Superior Occipital Gyrus	19	32	−86	24	4.64	0.000	0.020	0.000	1368
	Parietal	Precuneus/Superior Parietal Lobule	7	20	−66	52	3.96		0.232	0.000	
L	Parietal	Precuneus/Superior Parietal Lobule	7	−28	−70	32	3.86	0.009	0.319	0.000	359
	Temporal	Middle Occipital Gyrus	39	−36	−68	22	3.85		0.325	0.000	
	Occipital	Superior Occipital Gyrus	19	−36	−76	22	3.71		0.463	0.000	
R	Parietal	Temporoparietal Junction	40	44	−40	46	3.56	0.033	0.623	0.000	247

*BA, Brodmann area; L, Left; R, Right; FWE, family-wise error; a cluster level at the threshold of *p* < 0.05 (FWE corrected) with the voxel-level at the threshold of *p* < 0.001 (uncorrected). Cluster size is in voxels; voxel size is 2 × 2 × 2 mm^3^*.

#### ROI Analysis

Because the present study focused on the manner in which attentional orienting was influenced by self-referential processing based on activity in the dorsal and ventral frontoparietal networks, the results of the interactions in the bilateral SPL and right TPJ were further assessed using an ROI-based analysis. Figure 4 and Table 4 present the locations and patterns of the responses in all ROIs from which a beta value was extracted (i.e., the bilateral SPL and right TPJ). The beta values were analyzed with a 2 (cue: self- and neutral-arrow) × 2 (congruence: congruent and incongruent) repeated-measures ANOVA and revealed significant interactions in the bilateral SPL (left SPL: *F*_(1,23)_ = 29.456, *p* < 0.001, $\eta_{\text{p}}^{2}$ = 0.562; right SPL: *F*_(1,23)_ = 30.391, *p* < 0.001, $\eta_{\text{p}}^{2}$ = 0.569) and right TPJ (*F*_(1,23)_ = 14.202, *p* = 0.001, $\eta_{\text{p}}^{2}$ = 0.382; Table 4).

**Table 4:** ROI results.

Regions defined by locating local maxima
Region	Interaction *F*	Self Incon vs. Con	Neutral Incon vs. Con	Con Self vs. Neutral	Incon Self vs. Neutral
Left precuneus/SPL	29.456***	0.992	52.088***	9.273**	12.927**
Right precuneus/SPL	30.391***	2.309	35.870***	15.840**	13.082**
Right TPJ	14.202**	3.608	15.104**	7.411*	11.871**

ROIs represent previously examined areas that exhibited a significant interaction between the cue and congruence conditions in a 2 × 2 ANOVA; a cluster level at the threshold of *p* < 0.05 (FWE corrected) with the voxel-level at the threshold of *p* < 0.001 (uncorrected). Con: congruent; Incon: incongruent. *p < 0.05, **p < 0.01, ***p < 0.001.

A *post hoc* test revealed that the beta values were lower under the congruent condition than under the incongruent condition when using a neutral-arrow cue (left SPL: *p* < 0.001, right SPL: *p* < 0.001, and right TPJ:* p* = 0.01) but not when using a self-referential arrow cue (left SPL: *p* = 0.33, right SPL: *p* = 0.142, and right TPJ: *p* = 0.001). These findings are consistent with those of previous studies showing stronger activity under incongruent conditions compared to congruent conditions when using a neutral-arrow as a cue (Corbetta et al., 2008; Corbetta and Shulman, 2011). We also found higher beta values under the congruent condition (left SPL: *p* = 0.006, right SPL: *p* = 0.001, and right TPJ: *p* = 0.012) but lower ones under the incongruent condition (left SPL: *p* = 0.002, right SPL: *p* = 0.001, and right TPJ: *p* = 0.002) when a self-arrow cue was used but not when a neutral-arrow cue was used. These findings indicate that the neural activity underlying attentional orienting was influenced by self-referential processing under both the congruent and incongruent conditions. The ROI analysis also found a similar pattern in the active areas when using a control analysis with a relatively liberal threshold in the whole-brain analysis (Supplementary Figure S1, Table S3).

## Discussion

In the training task used in the present study, the participants responded more quickly and accurately to the arrows associated with “self” than to those associated with “other.” Previous studies investigating priority in the processing of self- vs. other-referential information have shown that participants respond more quickly and accurately to one’s own face than to another’s face (Keyes and Brady, 2010; Lv et al., 2015) and that stimuli (i.e., arrow or face) elicit a quicker response when associated with “self” than with “other” (Zhao et al., 2015). Likewise, the present study demonstrated that self-referential arrows had a higher processing priority than other-referential arrows.

Importantly, main effect analyses of fMRI data from the cueing task revealed significant activity in the left ACC when a self-arrow cue was used vs. when a neutral-arrow cue was used. A correlation analysis found that the self-arrow RT was facilitated by increased activity in the left ACC when comparing self- and neutral-arrows. The coordinate of the activity in the left ACC was consistent with previous studies (Holt et al., 2011; reviews in Hu et al., 2016), which demonstrated that the ACC/vmPFC are associated with self-referential processing. In particular, Sui et al. (2013) used the same technique as the present study to establish an association between either “self,” “friend,” or “other” and neutral geometric shapes (triangle, circle, or square). These authors found that the ACC/vmPFC region was more strongly activated in response to neutral shapes associated with “self” than to those associated with other words, regardless of the familiarity of the stimulus. The present study extended these findings by performing a direct comparison of brain activity between self- and neutral-arrow conditions. Given that the present results also revealed a significant correlation between the RT in self-referential processing and brain activation in the left ACC, we suggest that the contrasted brain activity in CMS likely reflected increases due to self-referential processing rather than decreases due to other-referential processing. Taken together, the present findings suggest that the arrow associated with “self” triggered self-referential processing during the cueing task due to the experiences of the participant in the training task.

Interaction analyses of the fMRI data indicated that there were different patterns of attentional orienting between the self- and neutral-arrow conditions under incongruent vs. congruent conditions. Previous studies have consistently reported greater degrees of neural activity in attentional networks under incongruent than congruent conditions when using a neutral-arrow cue (Corbetta et al., 2008; Corbetta and Shulman, 2011). Consistent with this, the present study observed more neural activity in the ventral and dorsal frontoparietal networks, including the right TPJ and the left/right SPL, under incongruent vs. congruent conditions when using a neutral-arrow cue. However, this difference was not seen between the incongruent and congruent conditions when using a self-arrow cue. Taken together, these findings suggest that the neural activity underlying attentional processing could be modulated based on the meaning of a cue. Accordingly, Özdem et al. (2016) observed increased activation in the TPJ under the incongruent condition compared to the congruent condition when the gaze change of a robot was believed to be controlled by a human as opposed to being pre-programmed. Moreover, in a behavioral study, Zhao et al. (2015) showed that self-referential arrow cues induced a similar pattern of attentional orienting with gaze cues after the association between non-predictive arrow cues and words (“self” and “other”) was established in a training task. These findings suggest that directional cues develop qualitatively different functions during attentional orienting due to self-referential processing.

The present findings extend those of a previous study (Zhao et al., 2015) by demonstrating that the neural activities associated with attentional orienting qualitatively rather than quantitatively differed between self- and neutral-arrow cues after an association between arrow cues and the “self” was established in the training task. It is possible that the pattern of neural activity elicited by self-arrow cues is similar to that elicited by social cues (i.e., gaze). Consistent with this proposition, Engell et al. (2010) contrasted incongruent and congruent conditions and found that qualitatively different patterns of brain activity emerged in the frontoparietal network when comparing arrow and gaze cues. Similarly, the present study showed that the regions within the dorsal and ventral frontoparietal networks, including the right TPJ, IFG and IPS, responded differentially to incongruent vs. congruent arrow cues but not to incongruent vs. congruent gaze cues. That is, there was greater neural activity under the incongruent condition compared to the congruent condition when using an arrow as a cue, but these networks were similarly recruited for the incongruent and congruent conditions when using gaze cues. A direct comparison of attentional orienting between gaze vs. arrow and self- vs. neutral-arrow conditions should be performed in future research, as it will be important to reach a firm conclusion regarding whether the neural pattern associated with attentional orienting during self-referential cues is similar to that elicited by gaze cues.

Furthermore, the interaction analyses of the fMRI data revealed that self-referential arrows influenced the neural activity underlying attentional orienting under both the incongruent and congruent conditions when a neutral-arrow cue was used as the baseline condition. The ROI analyses showed that, compared to the neutral-arrow cue conditions, the neural activity in the dorsal and ventral frontoparietal networks, including in the SPL and TPJ, in response to the self-arrow cue was reduced under the incongruent condition whereas this neural activity was enhanced under the congruent condition. Previous studies have consistently shown that self-referential processing aids in the effective capture of information useful for cognitive abilities, including attention and memory (for reviews, see Sui et al., 2015; Sui and Humphreys, 2017). For example, Zhao et al. (2015) found that self-referential cues were preferentially associated with a voice target in a cueing paradigm after the association between non-predictive cues and words (“self” and “other”) was established in a training task; therefore, the cueing effect is enhanced by self-referential cues for a voice target relative to a tone target but not by other-referential cues. Based such findings, it is possible that the present results reflect the priority of self-referential processing and its influence on attentional orienting during behavioral processes, which aids in the effective capturing of information. That is, the reduced neural activity associated with self-referential processing under the incongruent condition may indicate that participants can more rapidly disengage their attention from the cued location to capture a target, whereas the enhanced activity during self-referential processing under the congruent condition may show that attentional orienting was more strongly triggered by self-arrow cues than neutral-arrow cues and helped to capture the cued target.

However, there were no significant differences in the behavior elicited by the self-arrow and neutral-arrow cues under either the incongruent or the congruent condition in the present study. The design of the present cueing paradigm is relatively simple and may cause difficulties when attempting to distinguish behavioral differences; however, this paradigm can be effectively used to examine differences in neural activity following various cues during attentional orienting. Engell et al. (2010) used a cueing paradigm that is similar to the one used in the present study to examine differences between gaze and arrow cues. These authors found qualitative differences in the fMRI data between gaze and arrow cues, but no differences in the behavioral data were found under the congruent and incongruent conditions. It is possible that the different influences of self-referential processing on the neural activity associated with attentional orienting under the incongruent and congruent conditions shown in the present study would have been more evident behaviorally if they had been assessed using a more difficult paradigm; indirect evidence supports this idea (e.g., Friesen et al., 2004; Sui et al., 2009; Yan et al., 2016). For example, using an identification task, Sui et al. (2009) asked participants to identify whether a target at a left or right location was upright or inverted when using arrows after training trials were performed to associated the arrow cues with the words “self” and “friend”. There was a faster response under the incongruent condition when using self- vs. friend-arrow cues at a short SOA (250 ms), which indicates that participants might have been able to more rapidly disengage attention from the cued location to capture a target. Friesen et al. (2004) used a counterpredictive cueing paradigm in which the target was more likely (i.e., 75% of the trials) to appear at the location opposite to that of the cue direction, which is more demanding in terms of the top-down control of attention compared to a non-predictive paradigm. These authors found that attentional orienting was more strongly triggered by gaze cues than arrow cues under a short SOA condition and suggested that this aids in capturing a cued target. Based on the finding that the neural activity associated with attentional orienting was elicited by self-arrow cues in a manner similar to that elicited by gaze cues, it can be speculated that attentional orienting and attentional disengagement are more strongly triggered by self-arrow cues in a counterpredictive cueing paradigm. Future studies should directly investigate whether the influence of self-referential processing on the neural activity associated with attentional orienting can be quantified in behavioral terms using a difficult paradigm.

There were some limitations to the current study. First, this study contrasted incongruent vs. congruent conditions to investigate the influence of self-referential processing on attentional orienting. In previous studies, neutral cues (e.g., direct gaze) were manipulated as a baseline condition to examine the neural mechanisms underlying attentional orienting under incongruent and congruent conditions (e.g., Hietanen et al., 2006; Lockhofen et al., 2014; Joseph et al., 2015). However, compared to the use of non-directional arrows as neutral cues, the use of a direct gaze used as a neutral cue was perceived as directional rather than non-directional, which is problematic in terms of comparing arrow cues vs. social gaze cues (Engell et al., 2010). This study also contrasted incongruent vs. congruent conditions to examine differences in attentional orienting between arrow and social gaze cues. To determine whether the neural pattern associated with attentional orienting during the presentation of self-referential arrow cues was similar to the gaze pattern reported by Engell et al. (2010), the present study employed a design that contrasted incongruent vs. congruent conditions. Future research should investigate differences between self- and other-referential cues by comparing congruent vs. neutral and incongruent vs. neutral cues.

Second, the present study only examined the difference in the effect of attentional orienting on neural activity between self-referential and neutral-cues. Zhao et al. (2015) found that attentional orienting based on gaze cues could be inhibited by other-referential processing. Although a gaze cue can induce an enhanced cueing effect to a voice vs. a tone, after a facial gaze was associated with the other condition, the cueing effect to a voice target was inhibited by an other-referential gaze. Compared with self-referential processing to enhance attentional orienting, we speculated that other-referential processing might inhibit related information via a different mechanism. Thus, future research should compare neural activity between other-referential cues and neutral cues with respect to attentional orienting.

Finally, we did not monitor subjects’ eye movements. Friesen et al. (2004) monitored eye position and showed that the attention effects produced by non-predictive directional cues were not affected by a subject’s eye movements. Moreover, given that our results did not show significant activity in FEF regions, which are responsible for saccadic eye movements, this might reflect that the current findings did not depend on subjects’ eye movements. However, future research should use eye tracking and examine whether subjects’ eyes move towards the target before making a response.

## Conclusion

In the present study, neural activities in the CMS were enhanced when using self- vs. neutral-arrow cues in a cueing paradigm after the association between non-predictive cues (arrow) and words (“self”) were established in a training task. This finding suggests that the arrow associated with “self” triggered self-referential processing during the cueing task due to the experiences of the participant in the training task. Next, neural activities under the incongruent and congruent conditions were contrasted, revealing that qualitatively different patterns of attentional processing between self- and neutral-arrow cues emerged due to self-referential processing. It is possible that the neural activity associated with attentional orienting was elicited by self-referential arrow cues in manner similar to that elicited by social gaze stimuli (Engell et al., 2010). Furthermore, using a neutral-arrow cue as a baseline condition, the present study found that neural activity was reduced by self-referential processing under the incongruent condition but enhanced by self-referential processing under the congruent condition in the dorsal and ventral frontoparietal networks. These results suggest that when a stimulus is associated with the self, it modulates subsequent attentional neural processes. This process may be triggered by self-reference to automatically and effectively capture relevant information.

## Author Contributions

SZ and CL collected the data; SZ analyzed the data, prepared the figures and drafted the manuscript; SU made a substantial contribution to writing and revising the manuscript; and all authors participated in designing the experiments, contributed to manuscript development and read and approved the final manuscript.

## Conflict of Interest Statement

The authors declare that the research was conducted in the absence of any commercial or financial relationships that could be construed as a potential conflict of interest.

## Footnotes

^1^http://www.fil.ion.ucl.ac.uk/spm/software/spm12
