# Supplementary material for: The Influence of Self-Referential Processing on Attentional Orienting in Frontoparietal Networks
Source: Front Hum Neurosci. 2018 May 15;12:199. doi: 10.3389/fnhum.2018.00199 (PMC5962753; doi:10.3389/fnhum.2018.00199)
Supplement: Supplementary file 1 [file Table_1.DOCX]

**Table S1.** Supplement main effects of cue condition: self-arrow > neutral-arrow

| Side | Area | Region | BA | Coordinates | | | Z-value | P (FWE) | P (FWE) | P (uncorr) | Cluster size |
| --- | --- | --- | --- | --- | --- | --- | --- | --- | --- | --- | --- |
|  |  |  |  | x | y | z |  | (cluster level) | (peak level) | (peak level) |  |
| Exploratory whole-brain analysis | | | | | | | | | | | |
| L | Limbic | Anterior Cingulate | 24/32 | -16 | 44 | 12 | 3.78 | 0.780 | 0.343 | 0.000 | 17 |
| R | Parietal | Precuneus | 7 | 20 | -50 | 58 | 3.43 | 0.780 | 0.506 | 0.000 | 17 |
|  | Limbic | Anterior Cingulate | 32/33 | 18 | 30 | 16 | 3.43 | 0.834 | 0.711 | 0.000 | 11 |
|  | Parietal | Precuneus | 7 | -16 | -50 | 52 | 3.21 | 0.881 | 0.893 | 0.000 | 6 |

BA = Brodmann area; L=Left; R=Right; A voxel-level at the threshold of *p* < 0.001 (uncorrected) with a minimum cluster-level at the threshold of 5 voxels. Cluster size is in voxels; voxel size is 2×2×2 mm^3^.
