# Supplementary material for: The Influence of Self-Referential Processing on Attentional Orienting in Frontoparietal Networks
Source: Front Hum Neurosci. 2018 May 15;12:199. doi: 10.3389/fnhum.2018.00199 (PMC5962753; doi:10.3389/fnhum.2018.00199)
Supplement: Supplementary file 2 [file Table_2.DOCX]

**Table S2.** Supplement interactions between cue and congruence conditions

| Side | Area | Region | BA | Coordinates | | | Z-value | P (FWE) | P (FWE) | P (uncorr) | Cluster size |
| --- | --- | --- | --- | --- | --- | --- | --- | --- | --- | --- | --- |
|  |  |  |  | x | y | z |  | (cluster level) | (peak level) | (peak level) |  |
| Exploratory whole-brain analysis | | | | | | | | | | | |
| R | Occipital | Superior Occipital Gyrus | 19 | 32 | -86 | 24 | 4.64 | 0.000 | 0.020 | 0.000 | 1368 |
|  | Parietal | Precuneus/  Superior Parietal Lobule | 7 | 20 | -66 | 52 | 3.96 |  | 0.232 | 0.000 |  |
|  | *Occipital* | *Cuneus* | *18/19* | *2* | *-86* | *32* | *3.59* |  | *0.596* | *0.000* |  |
| L | Parietal | Precuneus/  Superior Parietal Lobule | 7/31 | -28 | -70 | 32 | 3.86 | 0.009 | 0.319 | 0.000 | 359 |
|  |  | Middle Temporal Gyrus | 39 | -36 | -68 | 22 | 3.85 |  | 0.325 | 0.000 |  |
|  |  | Superior Occipital Gyrus | 19 | -36 | -76 | 22 | 3.71 |  | 0.463 | 0.000 |  |
| *R* | *Parietal* | *Paracentral Lobule* | *4* | *2* | *-44* | *70* | *3.76* | *0.358* | *0.404* | *0.000* | *74* |
| *R* | *Frontal* | *Superior Frontal Gyrus* | *9* | *40* | *38* | *36* | *3.65* | *0.711* | *0.524* | *0.000* | *27* |
|  |  | *Middle Frontal Gyrus* | *46* | *46* | *38* | *28* | *3.30* |  | *0.877* | *0.000* |  |
| R | Parietal | Temporoparietal Junction | 40 | 44 | -40 | 46 | 3.56 | 0.033 | 0.623 | 0.000 | 247 |
| *R* | *Frontal* | *Inferior Frontal Gyrus* | *46* | *48* | *44* | *12* | *3.50* | *0.866* | *0.692* | *0.000* | *11* |
| *R* | *Frontal* | *Inferior Frontal Gyrus* | *9* | *46* | *8* | *34* | *3.47* | *0.602* | *0.721* | *0.000* | *39* |
| *R* | *Parietal* | *Precuneus* | *7* | *4* | *-78* | *52* | *3.18* | *0.913* | *0.945* | *0.000* | *6* |
| *L* | *Parietal* | *Superior Parietal Lobule* | *7* | *-26* | *-66* | *58* | *3.17* | *0.768* | *0.947* | *0.000* | *21* |

Regions are highlighted in ltalic text to supplement table 3.

BA = Brodmann area; L=Left; R=Right; A voxel-level at the threshold of *p* < 0.001 (uncorrected) with a minimum cluster-level at the threshold of 5 voxels. Cluster size is in voxels; voxel size is 2×2×2 mm^3^.
