# Supplementary material for: The Influence of Self-Referential Processing on Attentional Orienting in Frontoparietal Networks
Source: Front Hum Neurosci. 2018 May 15;12:199. doi: 10.3389/fnhum.2018.00199 (PMC5962753; doi:10.3389/fnhum.2018.00199)
Supplement: Supplementary file 3 [file Table_3.DOCX]

**Table S3.**

Supplement ROI results

| Regions defined by locating local maxima | | | | | |
| --- | --- | --- | --- | --- | --- |
| Region | Interaction *F* | Self Incon vs. Con | Neutral Incon vs. Con | Con Self vs. Neutral | Incon Self vs. Neutral |
| Right SOG(32,-86,24) | 28.029*** | 5.15* | 33.601*** | 6.08* | 27.235*** |
| Right Cuneus(2,-86,32) | 13.337*** | 6.873* | 9.057** | 8.445** | 7.229* |
| Right MTG(-36,-68,22) | 26.003*** | 0.943 | 49.167*** | 9.413** | 13.392*** |
| Right SOG(-36,-76,22) | 16.405*** | 1.12 | 32.353*** | 5.984* | 14.912** |
| Right Paracentral Lobule (2,-44,70) | 16.699*** | 9.648** | 7.801* | 11.139** | 6.541* |
| Right SFG(40,38,36) | 16.434*** | 5.497* | 11.188** | 7.616* | 8.821* |
| Right MFG (46,38, 28) | 12.089** | 5.412* | 8.729** | 5.474* | 8.938** |
| Right IFG(48,44,12) | 11.789** | 4.577* | 11.242** | 5.428* | 7.956* |
| Right IFG(46,8.34) | 12.98** | 2.927 | 11.527** | 1.938 | 14.308** |
| Right Preceneus (4,-78,52) | 21.197*** | 1.733 | 19.256*** | 7.695* | 9.673** |
| Right SPL(-26,-66,58) | 23.246*** | 1.198 | 28.065*** | 10.696** | 8.52** |

ROIs represent previously examined areas (Regions are highlighted in ltalic text in table S2) that exhibited a significant interaction between the cue and congruence conditions in a 2 × 2 ANOVA; A voxel-level at the threshold of *p* < 0.001 (uncorrected) with a minimum cluster-level at the threshold of 5 voxels. Con: congruent; Incon: incongruent.

***: *p <* 0.05, ****: *p <* 0.01, *****: *p <* 0.001.
