# Supplementary material for: The Influence of Self-Referential Processing on Attentional Orienting in Frontoparietal Networks
Source: Front Hum Neurosci. 2018 May 15;12:199. doi: 10.3389/fnhum.2018.00199 (PMC5962753; doi:10.3389/fnhum.2018.00199)
Supplement: Supplementary file 4 [file Image_1.PDF]

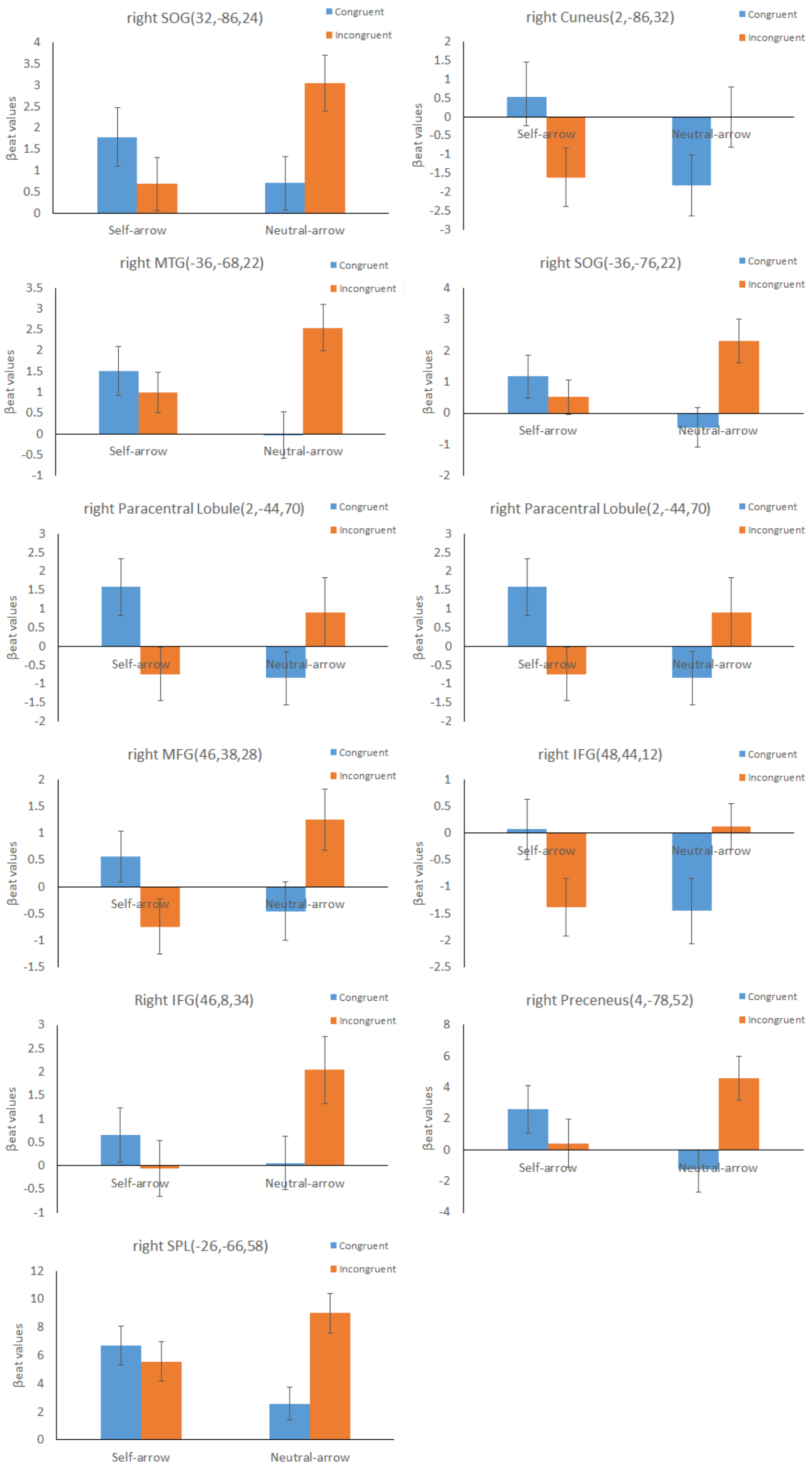

Figure S1. The figure shows mean beta values (± SE) in supplement regions; these areas are overlaid on the mean normalised structural MRI scans from all participants in the present study.
